# Anion-Dependent Synthesis of Cu(II) Complexes with 2-(1*H*-Tetrazol-5-yl)-1*H*-indole: Synthesis, X-Ray Structures, and Radical Scavenging Activity

**DOI:** 10.1155/2021/6736908

**Published:** 2021-12-21

**Authors:** Petr Halaš, Juraj Kuchár, Radovan Herchel

**Affiliations:** ^1^Department of Inorganic Chemistry, Faculty of Science, Palacký University Olomouc, 17. Listopadu 12, CZ-771 46 Olomouc, Czech Republic; ^2^Department of Inorganic Chemistry, Institute of Chemistry, Faculty of Science, Pavol Jozef Šafárik University in Košice, Moyzesova 11, SK-041 54 Košice, Slovakia

## Abstract

Two mononuclear Cu(II) complexes, [Cu(phen)_2_(HL)]ClO_4_·H_2_O·2DMF (**1**) and [Cu(phen)_2_(HL)_2_]·EtOH (**2**), comprising 1,10-phentantroline (phen) and 2-(1*H*-tetrazol-5-yl)-1*H*-indole ligand (H_2_L) ligands are reported. Analysis and characterization of the samples were performed using standard physicochemical techniques, elemental analysis, nuclear magnetic resonance, Fourier transform infrared spectroscopy, and UV-vis spectroscopy. Single-crystal X-ray crystallography revealed the formation of a pentacoordinate complex in **1** and a hexacoordinate complex in **2**, in which the anionic ligand HL^−^ has undergone monodentate coordination through the tetrazole unit. Furthermore, the crystal structure of H_2_L·MeOH is also discussed. The potential application of compounds **1** and **2** in bioinorganic chemistry was addressed by investigating their radical scavenging activity with the 2,2-diphenyl-1-picrylhydrazyl radical (DPPH) and the results were supported also by theoretical calculations.

## 1. Introduction

Reactive oxygen species (ROS) and their physiological effects have been studied extensively since their discovery circa 60 years ago [1]. These ROS can be divided into two groups: free oxygen radicals such as superoxide radical O_2_^·−^, hydroxyl radical OH**˙**, or organic peroxyl radicals ROO**˙** and nonradical ROS such as ozone O_3_, hydrogen peroxide H_2_O_2_, and singlet oxygen ^1^O_2_ [2]. As the name suggests, these molecules are very reactive and are partly responsible for oxidative stress in cells leading to lipid peroxidation [3] and damage to DNA and proteins [4, 5]. Their effect is generally considered toxic to the body and an increased level of these species has been linked to a number of pathologies such as inflammations [6], various cardiac diseases [7], and cancer [2].

The most common superoxide radical O_2_^·−^ is created in the electron transport chain, specifically in complexes I and III, as an unwanted by-product. Subsequently, this radical is released into the cytosol and in lesser degree to the mitochondrial matrix [8], where it then either reacts with nonenzymatic antioxidants, such as glutathione or ascorbic acid, or transforms into less damaging hydrogen peroxide or oxygen by metal-containing enzymes known as superoxide dismutases (SODs). The most common metal ions contained in these enzymes are Cu^2+^, Zn^2+^, and Mn^2+^; these are also directly involved in the enzymatic reaction due to their ability to transfer the unpaired electron from the superoxide radical without forming yet another highly reactive radical [9]. Klug-Roth and Rabani et al. have shown that copper ion cycles between oxidation states (I) and (II), as shown by reactions (1) and (2), creating either hydrogen peroxide or oxygen molecule [10].

(1) $$\begin{array}{c} {\text{Cu}\left(\text{II}\right)-\text{E }+\text{O}}_{2}^{\cdot -}{\text{Cu}\left(\text{I}\right)-\text{E }+\text{ O}}_{2} \end{array}$$

(2) $$\begin{array}{c} {\text{Cu}\left(\text{I}\right)-\text{E }+\text{O}}_{2}^{\cdot -}{+\;2\text{H}+\text{ Cu}\left(\text{I}\right)-\text{E }+\text{ H}}_{2}{\text{O}}_{2} \end{array}$$

To improve the health conditions of patients affected by diseases linked to the increased oxidative stress, many research groups have tried to prepare complex compounds of low molecular weight that would mimic the activity of SODs; unfortunately, SODs themselves cannot be administered, as they would not pass the cell membrane and are quickly metabolized by kidneys [11, 12]. In addition to the SOD-like activity of many copper compounds, many copper complexes are also known for their cytotoxic properties, most notably a group of ternary complexes called Casiopeínas. These copper complexes are made of substituted 1,10-phenanthroline or 2,2′-bipyridines and various anionic O,O- and N,O-ligands such as glycinate or acetylacetonate. The most prominent derivatives have been able to achieve values of IC_50_ in the range of low micromolar concentrations on several tumor cell lines [13, 14].

**Scheme 1:**
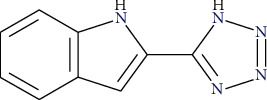
The scheme of 2-(1*H*-tetrazol-5-yl)-1*H*-indole (H_2_L) used as a ligand in this work.

To mimic the copper coordination sphere in Cu,Zn-SOD, which consists of 4 histidine ligands bound to a copper center by imidazole nitrogen atoms [15], we have decided to use a ligand containing tetrazole, 2-(1*H*-tetrazol-5-yl)-1*H*-indole (H_2_L) (Scheme 1). Tetrazole is also a five-membered nitrogen-containing ring similar to that of imidazole, but tetrazoles can readily release proton and have acidic properties similar to those of carboxylic acid.

Several complexes containing tetrazole have already exhibited cytotoxic properties, most notably a dimeric Pt(II) complex prepared by Komeda et al., which was more potent and much less toxic than gemcitabine on PANC-1 tumor transfected mice [16]. Indole moiety is also known to be present in a plethora of used chemotherapeutics, such as *Vinca* alkaloids or panobinostat, which was approved for use on multiple myeloma in 2015 [17]. New complexes containing ligands with indole and tetrazole moiety could therefore exhibit interesting biological properties. To the best of our knowledge, 2-(1*H*-tetrazol-5-yl)-1*H*-indole itself has not yet been tested for its cytotoxic properties and no complex comprising this ligand has been prepared yet. Additionally, inspired by the Casiopeínas group mentioned above, we chose 1,10-phenanthroline (phen) as a coligand for our synthesis. Thus, we herein report on synthesis of two copper complexes [Cu(phen)_2_(HL)]ClO_4_·H_2_O·2DMF (**1**) and [Cu(phen)_2_(HL)_2_]·EtOH (**2**), and their crystal structures as well as their scavenging activities.

## 2. Materials and Methods

All solvents and chemicals were purchased from various commercial sources and used without further purification. Elemental analysis was performed on the Thermo Scientific Flash 2000 analyzer. The infrared spectra of the complexes were measured on a Jasco FT/IR-4700 using ATR technique with a diamond plate in the range of 400–4000 cm^−1^. The ^1^H, ^13^C, and 2D NMR spectra of the ligand were measured on a 400 MHz Varian spectrometer. UV/Vis spectra were measured on Cintra 3030 (GBC Scientific Instruments) double beam spectrometer.

### 2.1. Synthesis

#### 2.1.1. Synthesis of 2-(1*H*-Tetrazol-5-yl)-1*H*-indole

The solution of 5 g (31 mmol) of 1*H*-indole-2-carboxylic acid in 25 ml of chloroform was mixed with 5 ml of thionyl chloride (SOCl_2_) and three drops of dimethylformamide (DMF). The reaction mixture was then refluxed for 2 hours. The cooled solution was then poured into a slurry of 20 ml of aqueous ammonia and 20 g of ice. The mixture was then stirred for 2 hours at room temperature, during which a large amount of yellow precipitate appeared. The solid product was filtered off, washed with water, and dried in a vacuum desiccator. The product, 1*H*-indole-2-carboxamide, was used in the next step without further purification (yield: 4.4 g (89%)).

6.3 g (39 mmol) of the previously prepared 1*H*-indole-2-carboxamide was added to 50 ml of phosphoryl chloride (POCl_3_). This mixture was refluxed for 30 minutes and subsequently it was poured onto 100 g of ice. The pH of the mixture was then adjusted to 8 by aqueous ammonia during which light brown product precipitated. This suspension was extracted 3 times with 50 ml of diethyl ether. The organic phase was dried over Na_2_SO_4_ and filtered and the solvent was removed to dryness on a rotatory evaporator. The light brown product, 1*H*-indole-2-carbonitrile, was dried in a vacuum desiccator and used in the next step without further purification (yield: 4.12 g (74%)).

4.12 g (29 mmol) of 1*H*-indole-2-carbonitrile was dissolved in 25 ml of DMF. To this was then added 3.77 g (58 mmol) of NaN_3_ and 1.55 g (29 mmol) of NH_4_Cl. This suspension was heated at 120°C for 18 hours. After it was cooled to room temperature, this mixture was poured into 100 ml of distilled water. The pH was adjusted to 1–2 with 2M HCl and the solution was extracted 3 times with 50 ml of ethyl acetate. The organic phase was washed with brine and dried over Na_2_SO_4_. After filtration, the solvent was removed on a rotatory evaporator to dryness. The resulting brown powder was recrystallized from methanol with a spoon of activated charcoal. The resulting off-white crystals of 2-(1*H*-tetrazol-5-yl)-1*H*-indole (H_2_L) were suitable for X-ray diffraction analysis (yield: 2.84 g (45%)).

Anal. Calcd. (%) for C_10_H_11_N_5_O (*M*_r_ = 217.1) corresponding to H_2_L^.^MeOH: C, 55.3; H, 5.1; N, 32.2. Found: C, 54.9; H, 4.9; N, 32.3. FT-IR (ATR, cm^−1^): 438w, 525w, 709w, 742s, 801m, 892w, 923m, 1005w, 1023w, 1083m, 1118w, 1137w, 1233m, 1249w, 1277w, 1338s, 1378s, 1414w, 1455w, 1509w, 1573w, 1618s, 2640w, 2687w, 2775w, 2832w, 2894w, 2964w, 3034w, 3111w, 3223s, 3362w, 3478w. ^1^H NMR (DMSO-*d*_6_) *δ* (ppm.): 7.04 (t, *J* = 7.80 Hz, 1 H, C4-H), 7.14 (s, 1 H, C2-H), 7.19 (t, *J* = 7.63 Hz, 1 H, C5-H), 7.47 (d, *J* = 8.22 Hz, 1 H, C6-H), 7.64 (d, *J* = 8.22 Hz, 1 H, C3-H), 12.13 (br. s., 1 H, N1-H). ^13^C NMR (DMSO-*d*_6_) *δ* (ppm.): 104.05, 112.66, 120.55, 121.58, 122.38, 124.01, 127.88, 137.82, 150.48.

#### 2.1.2. Synthesis of Compound **1**

42.7 mg (115 *μ*mol) of Cu(ClO_4_)_2_^.^6H_2_O was dissolved in 5 ml of DMF together with 45.6 mg (230 *μ*mol) of 1,10-phenanthroline monohydrate and 25 mg (115 *μ*mol) of H_2_L. Subsequently, 115 *μ*l of 1M aq. NaOH solution was added to deprotonate the ligand. The formation of green crystals suitable for an XRD analysis was observed upon diethyl ether vapors diffusion into the solution. The product was filtered off, washed with diethyl ether, and dried in a vacuum desiccator (yield: 56.8 mg (56%)).

Anal. Calcd. (%) for C_39_H_38_ClCuN_11_O_7_ (*M*_r_ = 871.8) corresponding to [Cu(phen)_2_(HL)]ClO_4_·H_2_O^.^2DMF: C, 53.7; H, 4.4; N, 17.7. Found: C, 53.6; H, 4.2; N, 17.3. FT-IR (ATR, cm^−1^): 427w, 521w, 620m, 660w, 721m, 755w, 847m, 1084s, 1145w, 1193w, 1223w, 1254w, 1308w, 1339w, 1362w, 1387w, 1424m, 1496w, 1518m, 1585w, 1604w, 1655s, 2924w, 3159w, 3380w.

#### 2.1.3. Synthesis of Compound **2**

46 mg (230 *μ*mol) of Cu(OAc)_2_^.^H_2_O was added to 10 ml of ethanol together with 91.2 mg (460 *μ*mol) of 1,10-phenanthroline monohydrate. After everything was dissolved, 50 mg (230 *μ*mol) of H_2_L was added to the reaction mixture. The solution was mixed to complete dissolution of the ligand and the clear solution was left to evaporate at room temperature, leading to the formation of green crystals suitable for an X-ray diffraction analysis. The resulting product was filtered off, washed with diethyl ether, and dried in a vacuum desiccator (yield: 67 mg (35%)).

Anal. Calcd. (%) for C_44_H_34_CuN_14_O (*M*_r_ = 838.4) corresponding to [Cu(phen)_2_(HL)_2_]·EtOH: C, 63.0; H, 4.1; N, 23.4. Found: C, 62.6; H, 3.8; N, 23.1. FT-IR (ATR, cm^−1^): 419w, 446w, 520w, 582w, 662w, 723s, 750m, 808w, 840m, 863w, 930w, 1035w, 1100w, 1143w, 1222w, 1306w, 1335s, 1363w, 1409m, 1423m, 1496w, 1514w, 1590w, 1625w, 3057w, 3345m.

### 2.2. X-Ray Crystallography

The data collection for H_2_L·MeOH (CSD number 2114450), **1** (CSD number 2114451), and **2** (CSD number 2114452) was carried out on SuperNova diffractometer from Rigaku OD equipped with Atlas2 CCD detector and Cu K*α* sealed tube as source. CrysAlisPro version 1.171.41.93a was used for the data collection and for the cell refinement, data reduction, and absorption correction [18]. The molecular structure of the prepared compounds was solved by SHELXT [19] and subsequent Fourier syntheses using SHELXL [20]. Anisotropic displacement parameters were refined for all non-H atoms. The hydrogen atoms were placed in calculated positions and refined riding on their parent C atoms with C–H (aliphatic) bond length of 0.98 Å and 0.99 Å and with C–H (aromatic) bond length of 0.95 Å in all three compounds. The hydrogen atoms of hydroxyl groups were also placed in calculated positions and refined riding on their parent O atoms with O–H bond length of 0.84 Å for H_2_L·MeOH and **2**. Hydrogen atoms of N–H group were found in a Fourier difference map and refined by riding model with N–H group bond length of 0.88 Å for H_2_L·MeOH, 0.89 Å for **1,** and 0.93 Å for **2**. Hydrogen atoms of water molecule in **1** were found in a Fourier difference map and refined by riding model with O–H group bond length of 0.827 and 0.910 Å. A geometric analysis was performed using SHELXL. DIAMOND [21] was used for molecular graphics.

### 2.3. DPPH Scavenging Activity

The DPPH scavenging assay was performed with some modifications according to a method reported by L. Tabrizi et al. [22]. In a cuvette, 150 *μ*l of 1 mM solution of DPPH in methanol was mixed with 150/300/450 *μ*l of 0.5 mM methanolic solution of a copper complex and the volume was adjusted to 3 ml with methanol. The solution was then mixed vigorously, and the absorbance was measured at 517 nm after 30 minutes. All experiments were carried out in triplicate. The resulting concentrations of the samples prepared this way were *c*_DPPH_ = 50 *μ*M and *c*_complex_ = 25/50/75 *μ*M.

The radical scavenging activity was then determined by the following equation:

(3) $$\begin{array}{c} A\left(\%\right)=\left(1-\frac{A}{A_{0}}\right)\times 100, \end{array}$$

where *A* is the measured absorbance of the sample and *A*_0_ is the absorbance of pure DPPH.

## 3. Results and Discussion

### 3.1. Synthesis and General Characterization

**Scheme 2:**
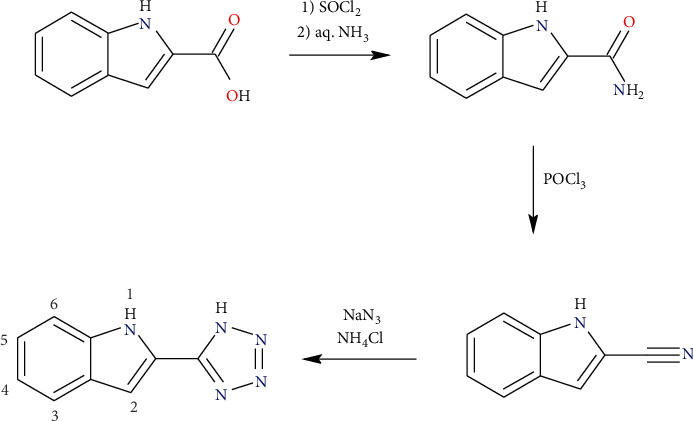
The three-step reaction scheme towards the preparation of 2-(1*H*-tetrazol-5-yl)-1*H*-indole (H_2_L).

First, 2-(1*H*-tetrazol-5-yl)-1*H*-indole (H_2_L) acting as a ligand was prepared by three-step chemical synthesis (Scheme 2), in which the 1*H*-indole-2-carboxylic acid was first converted to its amide using chlorination with SOCl_2_ and subsequent reaction with aq. ammonia. The resulting 1*H*-indole-2-carboxamide was dehydrated in the second step to a nitrile. The 1*H*-indole-2-carbonitrile was then transformed into a tetrazole derivative H_2_L with the help of *in situ* generated HN_3_ [23]. The formation of H_2_L was confirmed by the elemental analysis and ^1^H and ^13^C NMR (Figures S1 and S2). NMR spectra were compared with already published ones [24], which confirmed that synthesis of 2-(1*H*-tetrazol-5-yl)-1*H*-indole has been successful. In ^1^H NMR spectrum, we have additionally also observed a characteristic peak of CH_3_ group of methanol with a chemical shift *δ* = 3.14 ppm, which we at first assumed was residual solvent peak [25]. However, subsequent elemental analysis, as well as crystal structure determination, indeed showed that one molecule of methanol is present within the crystal structure, which is in accordance with the measured NMR spectra. Proton signals were then assigned with the help of COSY (Figure S3), HMBC (Figure S4), and HMQC (Figure S5) measurements. Doublets with chemical shifts *δ* = 7.47 and 7.64 ppm belong to protons in positions 6 and 3 (Scheme 2), respectively, whereas triplets with chemical shifts *δ* = 7.04 and 7.19 ppm belong to protons in positions 4 and 5, respectively. Singlet at 7.14 ppm is attributed to a proton in position 2. Lastly, broad singlet at 12.13 ppm belongs to a proton in an NH group of the indole ring. Tetrazole proton is not observed in the spectrum most likely due to its fast exchange in the solvent.

The coordination compounds [Cu(phen)_2_(HL)]ClO_4_·H_2_O^.^2DMF (**1**) and [Cu(phen)_2_(HL)_2_]·EtOH (**2**) were prepared by mixing the components Cu^II^ : phen : H_2_L in a molar ratio of 1 : 2 : 1, where copper perchlorate or copper acetate salts were utilized. In the synthesis of compound **1**, it was necessary to deprotonate the ligand H_2_L by adding an equimolar amount of 1 M aq. NaOH solution, while in the synthesis of compound **2**, anion of acetic acid originating from copper acetate acts as a base. The formation of suitable single crystals of **1** and **2** for the X-ray analysis was achieved either by diffusion of diethyl ether into the solution or by slow evaporation of the solvent. Also, it is worth mentioning that changing the molar ratio of Cu^II^ : phen : H_2_L in 1 : 1 : 1 resulted in products insoluble in common polar solvents. Infrared spectrum of H_2_L·MeOH comprises *ν*(O-H) and *ν*(N-H) stretching vibrations of methanol and H_2_L within 3100–3500 cm^−1^ range, while the aromatic *ν*(C-H) are observed around 3030 cm^−1^ (Figure Table S6). The aromatic system of H_2_L produced strong skeletal C=C/C=N/N=N ring vibrations visible at 1618–1338 cm^−1^ complemented by in-plane C-H and N-H bending vibrations at 1233–1024 cm^−1^. Below 1000 cm^−1^, the out-of-plane C-H/N-H and aromatic ring vibrations are present. The coordination compounds **1** and **2** produced complicated FT-IR spectra due to the mutual presence of two different N-heterocyclic aromatic ligands, H_2_L and phen, making the unequivocal assignment of the observed signals difficult (Figures S7–S9). Nevertheless, both complexes exhibit *ν*(N-H) stretching vibration of the indole of H_2_L in the range of 3300–3400 cm^−1^. Also, the different ratio of HL : phen in **1** and **2** is reflected in different relative transmittance of in-plane C/N-H and ring vibrations in 1655–1000 cm^−1^ region. The coordination of the heterocycles ligands is generally confirmed by shifting of characteristic vibrations. Thus, strong C=C/C=N vibrations of H_2_L at 1618 cm^−1^ have been significantly shifted downfield in both complexes **1** and **2** to 1518 and 1514 cm^−1^, respectively. Characteristic peaks of 1,10-phenanthroline can be found in spectra of both complexes at 736 and 850 cm^−1^, as well as around 1344, 1421, and 1503 cm^−1^ (Figure S9) [26]. The spectrum of **1** also comprises intensive peak at 1655 cm^−1^ that belongs to C=O stretching vibration of uncoordinated molecules of DMF. Furthermore, the presence of the noncoordinated perchlorate anion ClO_4_^−^ in **1** is clearly demonstrated by peak at 1084 cm^−1^ [27].

### 3.2. Description of the Crystal Structures

**Figure 1:**
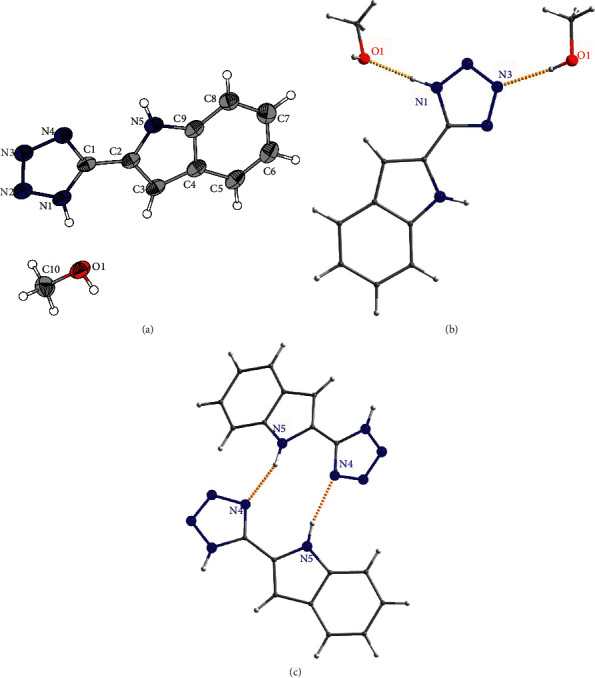
(a) ORTEP drawing of 50% probability with atom-numbering scheme for the asymmetric unit of H_2_L·MeOH. (b) The part of the crystal structure showing hydrogen bonds between methanol and H_2_L. (c) The part of the crystal structure showing intermolecular hydrogen bonds between H_2_L molecules.

First, the crystal structure of the ligand H_2_L·MeOH is discussed (Figure 1). The prepared single crystals belong to the monoclinic crystal system with the space group C 2/c (Table 1). As the data from elementary analysis and NMR spectroscopy suggest, a molecule of methanol is indeed present in the crystal structure, forming two types of hydrogen bonds with tetrazole rings. The first type is the hydrogen bond O1-H1⋯N3 having bond distance d(O1⋯N3) = 2.7952(27) Å (Figure 1(b)). The second type is formed between protonated tetrazole and methanol with d(N1⋯O1) = 2.6820 (22) Å (Figure 1(b)). There are also the intermolecular hydrogen bonds between H_2_L molecules formed between indole and tetrazole units with d(N5⋯N4) = 2.9466 (23) Å (Figure 1(c)). The detailed information about these hydrogen bonds is listed in Table 2.

**Figure 2:**
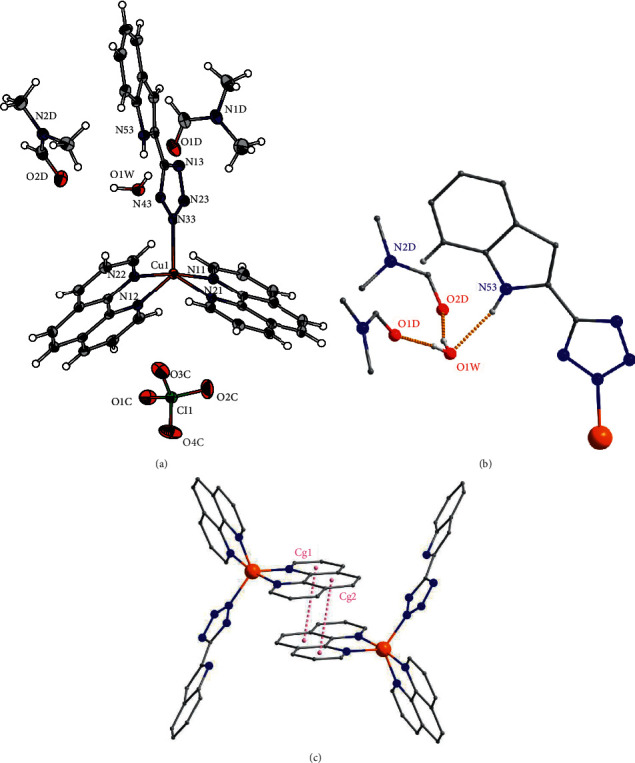
(a) ORTEP drawing of 50% probability with atom-numbering scheme for the asymmetric unit of **1**. (b) The part of the crystal structure showing hydrogen bonds between coordinated HL^−^ anion ligand, water, and DMF molecules. (c) The part of the crystal structure showing the formation of supramolecular dimers through *π*-*π* stacking, the hydrogen atoms were omitted for the sake of clarity.

Compound **1** crystallized in a triclinic crystal system with a space group P – 1 (Table 1). The asymmetric unit of **1** contains a [Cu(phen)_2_(HL)] complex, perchlorate anion, two molecules of DMF, and one molecule of water. The copper atom is coordinated by two phen ligands in bidentate fashion, and the anionic HL^−^ ligand is coordinated through the nitrogen atom of the tetrazole unit, with the respective Cu-N distances listed in Table 3. Thus, the coordination number is 5 for {CuN_5_} chromophore, and the Addison parameter [28] is equal to 0.83, which means the coordination polyhedron is close to a trigonal bipyramid, as it is also evident in Figure 2. The shortest distance between oxygen of perchlorate anion and copper atom is d (Cu1⋯O3C) = 3.9293 (17) Å, which means that the perchlorate anion is not coordinated. The cocrystalized solvents form net of the hydrogen bonds together with the indole part of the anionic ligand HL (Figure 2(b) and Table 2). Both DMF molecules form O-H⋯O hydrogen bonds with the following donor-acceptor distances: d (O1W⋯O1D) = 2.7378 (19) Å and d (O1W⋯O2D) = 2.7651 (18) Å. The next hydrogen bond is formed between the oxygen of water molecule and N-H group of indole with d(N53⋯O1W) = 2.8660 (19) Å. Furthermore, there is *π*-*π* stacking interaction within the crystal structure of **1** in which 1,10-phenanthroline ligands are involved and the distance between their centroids is of 3.6182 Å (Figure 2(c) and Table 4).

**Figure 3:**
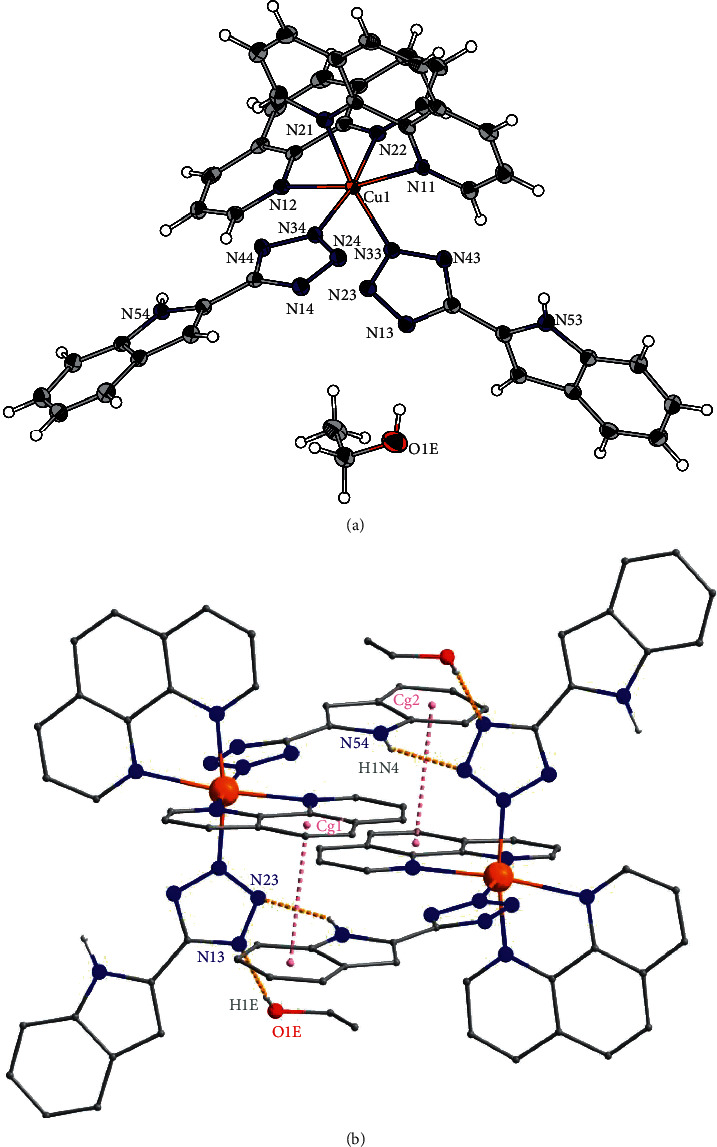
(a) ORTEP drawing of 50% probability with atom-numbering scheme for the asymmetric unit of **2**. (b) The part of the crystal structure showing the formation of supramolecular dimers through the hydrogen bonds and *π*-*π* stacking, only the hydrogen atoms bonded to nitrogen and oxygen are shown.

Crystal system of compound **2** was also triclinic with a space group P–1. The asymmetric unit of **2** contains a neutral complex [Cu(phen)_2_(HL)_2_] and a molecule of ethanol (Figure 3). The copper atom is coordinated by two bidentate phen ligands and two monodentate anionic HL^−^ ligands, thus forming {CuN_6_} chromophore with the respective Cu-N distances listed in Table 3. Due to Jahn-Teller effect, complex [Cu(phen)_2_(HL)_2_] of **2** shows elongated square-bipyramidal geometry, in which the axial positions are occupied by nitrogen atoms of two phen ligands (Figure 3(a)). Moreover, the formation of supramolecular dimers is observed in the crystal structure of **2**. These supramolecular dimers are stabilized by hydrogen bonds between nitrogen atom of tetrazole ring and protonated nitrogen of indole of the second complex with following donor-acceptor distances: d(N54⋯N23) = 3.098 (2) Å (Figure 3(b) and Table 2). Furthermore, the supramolecular dimer is additionally stabilized by *π*-*π* stacking interactions formed by neighboring 1,10-phenanthrolines and indoles with the shortest distance between benzene and indole centroids of 3.5806 (12) Å (Table 4 and Figure 3(b)). Lastly, a hydrogen bond is also present between nitrogen atom of tetrazole unit and ethanol molecule with d(O1E···N33) = 2.939 (2) Å (Figure 3(b)).

### 3.3. Radical Scavenging Activity

Spectroscopic determination of DPPH radical quenching is one of the most widely used methods which readily and reliably provide information about the radical scavenging activity of studied compounds. In our case, this represents a measure of the ability of studied complexes to scavenge detrimental radicals present in the intracellular environment, such as the aforementioned OH**˙** radical.

**Figure 4:**
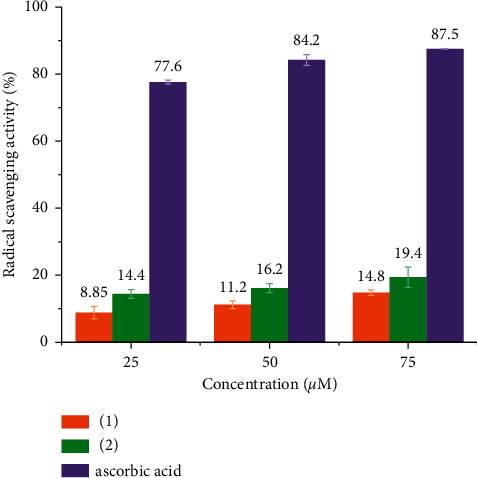
DPPH radical scavenging activity of complexes **1** and **2** and ascorbic acid for three different concentrations.

The DPPH scavenging activity for **1** and **2** was measured by UV-Vis spectroscopy at 517 nm in triplicate and the values were averaged for each concentration (Figure 4). Conveniently, the studied complexes show no significant absorption in this region (Figures S10 and S11). Evidently, both prepared complexes **1** and **2** possess DPPH radical scavenging activity, which increases with the increasing concentration of the copper complex (Figure 4). For comparison, we also measured the DPPH radical scavenging activity of ascorbic acid, which is much larger than those of both of our complexes. Compound **2** shows slightly higher activity than complex **1**. As the composition of both complexes is very similar, a possible reason for activity discrepancy between complexes **1** and **2** might be caused by the difference in their coordination polyhedra. Different geometries produce different ligand fields, which in turn affect energetic levels and splitting of d-orbitals. This consequently affects both the kinetic and thermodynamic behavior of the DPPH quenching reaction [22, 29, 30].

To elucidate possible reaction mechanism of the antioxidant activity of **1** and **2**, we performed theoretical calculations at DFT level of theory for complexes [Cu(phen)_2_(HL)]^+^ of **1** and [Cu(phen)_2_(HL)_2_] of **2**. Herein, ORCA 5.0 computation package [31, 32] was utilized together with *ω*B97M-D4 range-separated hybrid functional [33–36]. The triple-zeta bases sets def2-TZVP(-f) were used for all atoms [37] and the calculations were speeded up by using def2/J auxiliary basis [38] and RIJCOSX approximation [39–41]. As the experimental data were acquired with methanolic solutions, the geometry optimizations of the respective complexes were done with the conductor-like polarizable continuum model (C-PCM) using parameters for methanol solvent [42, 43]. The thermochemistry data were calculated as implemented in ORCA at 298.15 K and Gibbs free energies were corrected by where the factor of 1.89 kcal/mol is due to the change in standard state from gas phase to solution phase [44].

First, the hydrogen atom transfer (HAT) mechanism was evaluated with the help of the following reactions:

  [Cu(phen)_2_(HL)]^+^ ↔ [Cu(phen)_2_(L)]^+·^ + H^·^ (Δ_r_*G* = 87.13 kcal/mol)
  [Cu(phen)_2_(HL)_2_] ↔ [Cu(phen)_2_(HL) (L)]^·^ + H^·^ (Δ_r_*G* = 85.68 kcal/mol)

Next, we considered single electron transfer (SET) resulting in the solvated electron release/absorption as written here:

  [Cu(phen)_2_(HL)]^+^ + CH_3_OH ↔ [Cu(phen)_2_(L)]^2+^ + CH_3_OH^·−^ (Δ_r_*G* = 125.92 kcal/mol)
  [Cu(phen)_2_(HL)_2_] + CH_3_OH ↔ [Cu(phen)_2_(HL)_2_]^+^ + CH_3_OH^·−^ (Δ_r_*G* = 144.18 kcal/mol)
  [Cu(phen)_2_(HL)]^+^ + CH_3_OH^·−^ ↔ [Cu(phen)_2_(L)] + CH_3_OH (Δ_r_*G* = −87.26 kcal/mol)
  [Cu(phen)_2_(HL)_2_] + CH_3_OH^·−^ ↔ [Cu(phen)_2_(HL)_2_]^−^ + CH_3_OH (Δ_r_*G* = −81.58 kcal/mol)

Finally, the last evaluated mechanism is proton loss (PL), where the deprotonation of the nitrogen atom of the indole part of HL^−^ was considered:

  [Cu(phen)_2_(HL)]^+^ + CH_3_OH ↔ [Cu(phen)_2_(L)] + CH_3_OH_2_^+^ (Δ_r_*G* = 52.22 kcal/mol)
  [Cu(phen)_2_(HL)_2_] + CH_3_OH ↔ [Cu(phen)_2_(HL)(L)]^−^ + CH_3_OH_2_^+^ (Δ_r_*G* = 52.17 kcal/mol)

Evidently, the only spontaneous reaction (exergonic reactions) is attributed to SET in which the electron is donated to the complexes, which resulted in the reduction of Cu^II^ to Cu^I^. Moreover, the Δ_r_*G* = −87.26 kcal/mol for [Cu(phen)_2_(HL)]^+^ of **1** and the value of Δ_r_*G* = −81.58 kcal/mol for [Cu(phen)_2_(HL)_2_] of **2** are similar in agreement with the comparable radical scavenging activity of these compounds.

## 4. Conclusions

The impact of different copper salts on the preparation of metal complexes with 2-(1*H*-tetrazol-5-yl)-1*H*-indole ligand (H_2_L) was investigated. The single-crystal X-ray analysis confirmed formation of pentacoordinate [Cu(phen)_2_(HL)]ClO_4_·H_2_O·2DMF (**1**) and hexacoordinate [Cu(phen)_2_(HL)_2_]·EtOH (**2**) compounds. In both compounds, the anionic HL^−^ ligand acts as a monodentate N-donor ligand attached to the central atom through the tetrazole unit. The investigation of the interaction of these complexes with DPPH radical in their methanolic solution revealed moderate radical scavenging activity. The subsequent theoretical DFT calculations proposed that the dominant mechanism is the single electron transfer to the studied complexes.

## Figures and Tables

**Table 1:** Crystallographic data and details of structure refinement of the H_2_L ligand and coordination compounds **1** and **2**.

Compound	H_2_L·MeOH	**1**	**2**
Empirical formula	C_10_H_11_N_5_O	C_39_H_38_ClCuN_11_O_7_	C_44_H_34_CuN_14_O
Formula weight	217.24	871.79	838.39
T (K)	95	95	95
Crystal system, space group	Monoclinic, C 2/c	Triclinic, P–1	Triclinic, P–1
Unit cell dimensions			
a (Å)	22.6910 (14)	8.7031 (1)	11.9157 (5)
b (Å)	7.0280 (3)	14.9274 (2)	13.0812 (6)
c (Å)	13.5206 (7)	15.1670 (2)	13.3817 (7)
*α* (°)	90	93.267 (1)	68.410 (5)
*β* (°)	100.504 (5)	97.193 (1)	71.957 (4)
*γ* (°)	90	102.144 (1)	89.768 (4)
*V* (Å^3^)	2120.0 (2)	1904.09 (4)	1829.80 (16)
*Z* (*D*_*c*_/g cm^−3^)	8, 1.361	2, 1.521	2, 1.522
Absorption coefficient (mm^−1^)	0.783	2.024	1.331
Crystal size (mm)	0.37 × 0.16 × 0.04	0.36 × 0.21 × 0.08	0.16 × 0.10 × 0.06
*F* (000)	912.0	902.0	866.0
Θ range for data collection (°)	3.96 ≤ *θ* ≤ 74.66	2.95 ≤ *θ* ≤ 74.07	3.66 ≤ *θ* ≤ 74.65
Index ranges (*h*, *k*, *l*)	−25 ≤ *h* ≤ 27	−10 ≤ *h* ≤ 10	−14 ≤ *h* ≤ 14
	−8 ≤ *k* ≤ 8	−18 ≤ *k* ≤ 18	−11 ≤ *k* ≤ 16
	−16 ≤ *l* ≤ 7	−12 ≤ *l* ≤ 18	−16 ≤ *l* ≤ 16
Reflections collected/unique (*R*_int_)	3624/2097 (0.0205)	13771/7499 (0.0183)	12531/7230 (0.0261)
Data/restraints/parameters	2097/0/147	7499/0/536	7230/0/543
Goodness-of-fit on *F*^2^	1.094	1.069	1.019
Final *R* indices [*I* > 2*σ* (*I*)]	*R *_1_ = 0.0613, *wR*_2_ = 0.1619	*R *_1_ = 0.0310, *wR*_2_ = 0.0819	*R *_1_ = 0.0374, *wR*_2_ = 0.0925
*R* indices (all data)	*R *_1_ = 0.0667, *wR*_2_ = 0.1669	*R *_1_ = 0.0334, *wR*_2_ = 0.0842	*R *_1_ = 0.0457, *wR*_2_ = 0.0980
Largest peak and hole/e Å^−3^	0.595/−0.396	0.482/−0.503	0.835/−0.656

**Table 2:** Hydrogen bonds (Å) and angles (°) for H_2_L·MeOH, **1** and **2**.

D-H···A	d(D-H)	d(H···A)	d(D···A)	<(D-H···A)
H_2_L·MeOH^a^				
N1-H1N1⋯O1	0.89	1.79	2.682 (2)	174.3
N5-H1N5⋯N4^i^	0.91	2.07	2.947 (2)	162.3
O1-H1⋯N3^ii^	0.84	1.97	2.795 (2)	166.2

**1**				
N53-H1N5⋯O1W	0.88	2.00	2.8660 (18)	166.9
O1W-H1O1⋯O1D	0.83	1.91	2.7377 (18)	173.7
O1W-H2O1⋯O2D	0.91	1.87	2.7651 (18)	167.1

**2 ** ^b^				
N53-H1N3⋯N24^i^	0.94	2.33	3.144 (2)	143.9
N53-H1N3⋯N14^i^	0.94	2.69	3.388 (2)	131.6
N54-H1N4⋯N23^ii^	0.93	2.31	3.098 (2)	143.0
O1E-H1E···N13	0.84	2.12	2.939 (2)	164.1

Symmetry transformations used to generate equivalent atoms. ^a^i: −*x* + 1, y, −*z* + 3/2; ii: *x*, −*y* + 1, *z*−1/2, ^b^i: −*x* + 1, −*y* + 2, −*z* + 1; ii: −x + 1, −*y* + 1, −*z* + 1.

**Table 3:** Selected interatomic parameters (Å, °) for coordination compounds **1** and **2**.

[Cu(phen)_2_(HL)]ClO_4_·H_2_O·2DMF (**1**)		[Cu(phen)_2_(HL)_2_]·EtOH (**2**)	
Cu1-N11	2.0069 (13)	Cu1-N11	2.3624 (15)
Cu1-N12	2.0909 (13)	Cu1-N12	2.3541 (15)
Cu1-N33	2.0412 (13)	Cu1-N33	2.0528 (16)
Cu1-N21	2.1062 (14)	Cu1-N34	2.0146 (15)
Cu1-N22	2.0043 (13)	Cu1-N21	2.0770 (16)
N22-Cu1-N11	171.66 (5)	Cu1-N22	2.0571 (16)
N22-Cu1-N33	95.48 (5)	N21-Cu1-N11	75.29 (6)
N11-Cu1-N33	92.82 (5)	N22-Cu1-N11	96.58 (6)
N22-Cu1-N12	81.35 (5)	N33-Cu1-N11	96.23 (6)
N11-Cu1-N12	94.95 (5)	N34-Cu1-N11	91.00 (6)
N33-Cu1-N12	122.04 (5)	N21-Cu1-N12	95.47 (6)
N22-Cu1-N21	94.09 (5)	N22-Cu1-N12	75.66 (6)
N11-Cu1-N21	81.13 (5)	N33-Cu1-N12	92.62 (6)
N33-Cu1-N21	118.86 (5)	N34-Cu1-N12	95.82 (6)
N12-Cu1-N21	119.09 (5)	N22-Cu1-N21	87.20 (6)
		N12-Cu1-N11	168.39 (5)
		N33-Cu1-N21	171.18 (6)
		N34-Cu1-N21	88.34 (6)
		N33-Cu1-N22	91.42 (6)
		N34-Cu1-N22	169.95 (6)
		N34-Cu1-N33	94.33 (6)

**Table 4:** Short ring interactions with respective distances (Å) and angles (°) for **1** and **2**^*a*^.

Cg(I)	Cg(J)	d(Cg(I)···Cg(J))	d(CgI···Perp)	d(CgJ···Perp)	*γ*
**1 ** ^b^					
Cg1	Cg2 (1 − *x*, 1 − *y*, 1 − *z*)	3.6182 (10)	3.4624 (7)	3.5106 (7)	16.9
Cg2	Cg1 (1 − *x*, 1 − *y*, 1 − *z*)	3.6182 (10)	3.5107 (7)	3.4623 (7)	14.0

**2 ** ^c^					
Cg1	Cg2 (1 − *x*, 1 − *y*, 1 − *z*)	3.5806 (12)	3.3970 (8)	3.5078 (8)	18.4
Cg2	Cg1 (1 − *x*, 1 − *y*, 1 − *z*)	3.5806 (12)	3.5078 (8)	3.3970 (8)	11.6

^a^Cg = centroid, CgI···Perp = perpendicular distance of Cg(I) on ring J; CgJ···Perp = perpendicular distance of Cg(J) on ring I; *γ* = angle Cg(I) ⟶ Cg(J) vector and normal to plane J. ^b^ Cg1 is centroid of ring defined by N12-C12-C22-C32-C42-C122 atoms; Cg2 is centroid of ring defined by C42-C52-C62-C72-C112-C122 atoms. ^c^Cg1 is centroid of ring defined by C42-C52-C62-C72-C112-C122 atoms; Cg2 is centroid of ring defined by C44-C54-C64-C74-C84-C94 atoms.

## Data Availability

The data used to support the findings of this study are included within the article and the supplementary information file.

## Supplementary Materials

Supplementary Materials Figures S1–S5: NMR spectra of H_2_L. Figures S6–S9: FT-IR spectra of H_2_L, phen, and **1** and **2**. Figures S10 and S11: UV-VIS spectra of **1** and **2**. *XYZ* coordinates of DFT-optimized molecular geometries.
