# Supplementary material for: Anion-Dependent Synthesis of Cu(II) Complexes with 2-(1H-Tetrazol-5-yl)-1H-indole: Synthesis, X-Ray Structures, and Radical Scavenging Activity
Source: Bioinorg Chem Appl. 2021 Dec 21;2021:6736908. doi: 10.1155/2021/6736908 (PMC8714388; doi:10.1155/2021/6736908)
Supplement: Supplementary Materials — Figures S1–S5: NMR spectra of H2L. Figures S6–S9: FT-IR spectra of H2L, phen, and 1 and 2. Figures S10 and S11: UV-VIS spectra of 1 and 2. XYZ coordinates of DFT-optimized molecular geometries. [file 6736908.f1.docx]

**Supporting Information**

**Anion-dependent synthesis of Cu(II) compoundes with 2-(1H-tetrazol-5-yl)-1H-indole: Synthesis, X-ray structures, and radical scavenging activity**

Petr Halaš*^a^*, Juraj Kuchár*^a,b^*, Radovan Herchel*^a,*^*

*^a^* Department of Inorganic Chemistry, Faculty of Science, Palacký University Olomouc, 17. listopadu 12, CZ-771 46 Olomouc, Czech Republic

*^b^* Department of Inorganic Chemistry, Institute of Chemistry, Faculty of Science, P.J. Šafárik University in Košice, Moyzesova 11, SK-041 54 Košice, Slovakia

**List of supporting information**

**Figure S1.** 1H NMR spectrum of 2-(1*H*-tetrazol-5-yl)-1*H*-indole (H_2_L).

**Figure S2.** 13C NMR spectrum of 2-(1*H*-tetrazol-5-yl)-1*H*-indole (H_2_L).

**Figure S3.** COSY NMR spectrum of 2-(1*H*-tetrazol-5-yl)-1*H*-indole (H_2_L).

**Figure S4.** HMBC NMR spectrum of 2-(1*H*-tetrazol-5-yl)-1*H*-indole (H_2_L).

**Figure S5.** HMQC NMR spectrum of 2-(1*H*-tetrazol-5-yl)-1*H*-indole (H_2_L).

**Figure S6.** FT-IR spectrum of 2-(1*H*-tetrazol-5-yl)-1*H*-indole (H_2_L).

**Figure S7.** FT-IR spectrum of compound **1**.

**Figure S8.** FT-IR spectrum of compound **2**.

**Figure S9.** Comparison of FT-IR spectra of H_2_L, phen, and compounds **1** and **2**.

**Figure S10.** UV-Vis spectrum of compound **1** in MeOH solution (10 µM).

**Figure S11.** UV-Vis spectrum of compound **2** in MeOH solution (10 µM).

XYZ coordinates of the optimized geometries obtained by DFT calculations.

**Figure S1.** 1H NMR spectrum of 2-(1*H*-tetrazol-5-yl)-1*H*-indole (H_2_L) in DMSO-*d_6_*.

**Figure S2.** 13C NMR spectrum of 2-(1*H*-tetrazol-5-yl)-1*H*-indole (H_2_L) in DMSO-*d_6_*.

**Figure S3.** COSY NMR spectrum of 2-(1*H*-tetrazol-5-yl)-1*H*-indole (H_2_L).

**Figure S4.** HMBC NMR spectrum of 2-(1*H*-tetrazol-5-yl)-1*H*-indole (H_2_L).

**Figure S5.** HMQC NMR spectrum of 2-(1*H*-tetrazol-5-yl)-1*H*-indole (H_2_L).

**Figure S6.** FT-IR spectrum of 2-(1*H*-tetrazol-5-yl)-1*H*-indole (H_2_L).

**Figure S7.** FT-IR spectrum of compound **1**.

**Figure S8.** FT-IR spectrum of compound **2**.


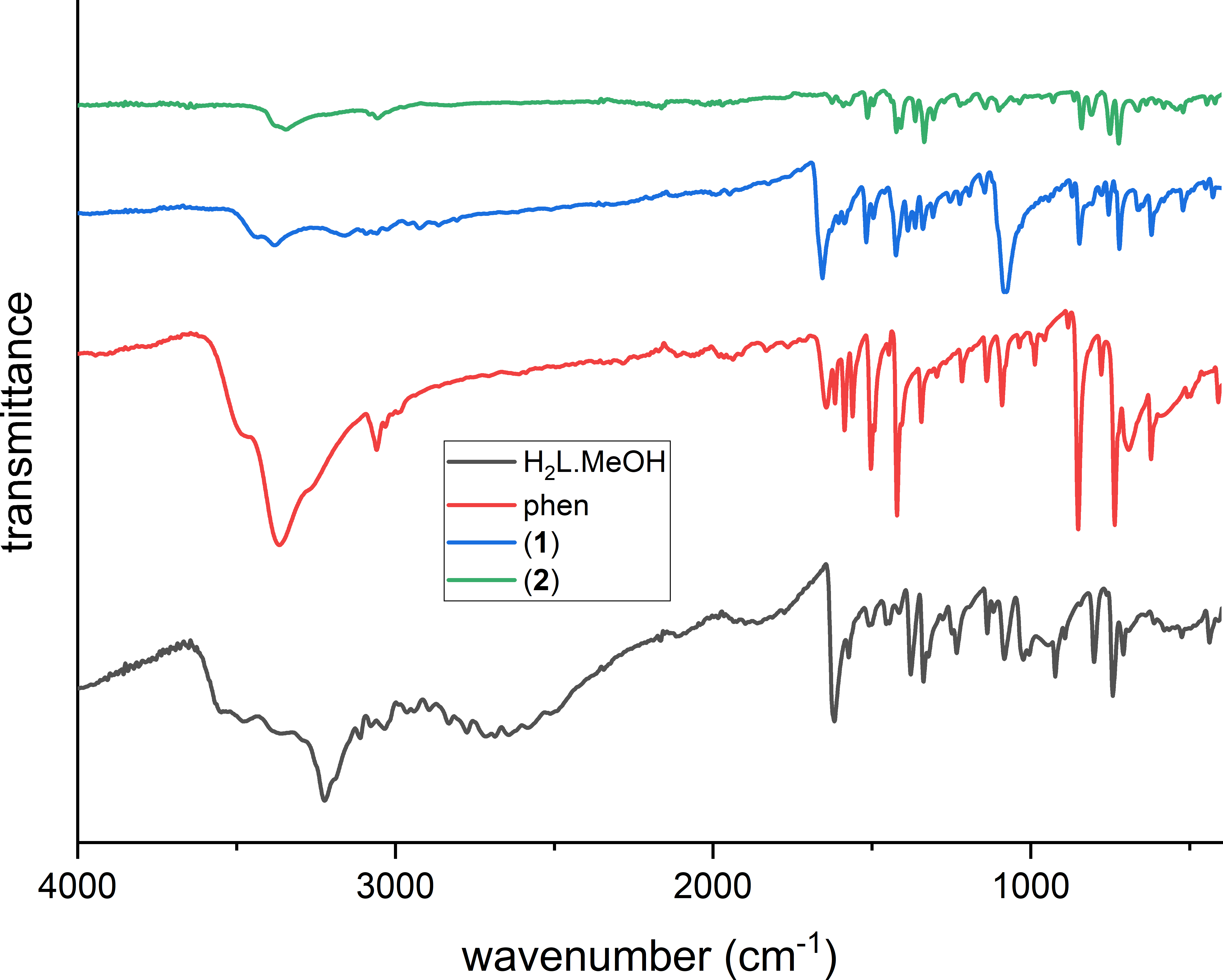


**Figure S9.** Comparison of FT-IR spectra of H_2_L, phen, and compounds **1** and **2**.

**Figure S10.** UV-Vis spectrum of compound **1** in MeOH solution (10 µM).

**Figure S11.** UV-Vis spectrum of compound **2** in MeOH solution (10 µM).

**List of XYZ coordinates calculated with** ωB97M-D4/def2-TZVP(-f)/C-PCM(CH_3_OH)

**[Cu(phen)_2_(HL)]^+^; multiplicity 2**

Cu 2.82043802126343 3.76194771611901 7.40628465281034

C 5.28198771443698 4.91958795129242 8.66789167059476

H 5.79154494638382 4.45192999785474 7.83800807699400

C 3.68734861740433 1.49089963264848 9.52023321357363

H 4.36328701507235 2.23107354112844 9.92743511781015

C 4.85916902451285 4.23575210215092 3.92901897696828

C 5.99097367351739 5.61188031506237 9.65452680689501

H 7.06580154339214 5.67880530564100 9.58447478991878

C 3.58246797212564 0.22296813942812 10.10698180133247

H 4.18390755341187 -0.01429171862527 10.97149398855385

C 5.40323350773913 3.76248936118464 2.66651121356012

C 5.30358056592255 6.19232746391333 10.68874976699131

H 5.82331849375110 6.73529224276137 11.46653824639490

C 2.71686115519017 -0.69088343944546 9.56616766164436

H 2.61194561387741 -1.67949231422800 9.99236423226035

C 6.11835349576128 4.43133749468611 1.71419212158246

H 6.40649315160087 5.46750501546110 1.75477975545953

C 3.90323883667136 6.07932636833443 10.74102938418113

C 1.95332238094604 -0.33676690328222 8.43954550676258

C 6.40064307604621 3.48719555343611 0.67337435587788

C 3.10100027045990 6.65043574682576 11.78661639224209

H 3.60113836537967 7.19836403405836 12.57317718442392

C 1.02443632948365 -1.22993987053038 7.80545581327268

H 0.90768297142830 -2.22351311712903 8.21594035266984

C 7.08426093427325 3.55734052571016 -0.54826143053039

H 7.53610513039439 4.48625435984018 -0.87058093100465

C 1.75970337846067 6.50567718989117 11.78761307660950

H 1.15615130590256 6.93510467823950 12.57526959801301

C 0.31859197122885 -0.84077401537916 6.72418825017905

H -0.37865022583859 -1.51314503085215 6.24374156493524

C 7.17064660247834 2.42492990610675 -1.32935978591517

H 7.69503163510083 2.46371210345458 -2.27453180323989

C 1.09622891493819 5.77708093847859 10.74269997034544

C 0.47295521535029 0.47949062441970 6.18033224684927

C 6.58641477914916 1.21199900178321 -0.91856775025817

H 6.67200415114113 0.34149923763944 -1.55485802643740

C -0.29671102475292 5.59069211961775 10.69081656642754

H -0.92063049976989 6.01046965794414 11.46824392371270

C -0.23677848646896 0.93610402987581 5.05653128824979

H -0.93720113733352 0.27472153326831 4.56478077011040

C 5.90752646975814 1.11220603003739 0.27748218057671

H 5.45697660253448 0.18203367179718 0.59599144450804

C -0.84206803281680 4.87850388484580 9.65511299447569

H -1.90665023224661 4.71548349696594 9.58299498527050

C -0.03131040251110 2.21124617144416 4.59695295984039

H -0.55697295137834 2.59244231325980 3.73496795464805

C 5.82313683668621 2.26119381276476 1.06485112046477

C 0.00191910826457 4.35216607115156 8.66933931276181

H -0.40460101770448 3.78605329717681 7.84287785764398

C 0.87985332609470 3.03405697474752 5.26431222005608

H 1.06760580102832 4.04548021141252 4.93224319345025

C 1.85278875689525 5.21519659292398 9.71449000380646

C 1.36545088893348 1.36779515049670 6.78240581923995

C 3.28025604268293 5.36939891088866 9.71531515552820

C 2.12648036208879 0.95129386599881 7.93035134135302

N 3.96858137090279 4.80260460114977 8.69882232488868

N 2.98515056097328 1.84540386202523 8.46463992550356

N 3.89097703638694 4.28421615046915 5.77637905928689

N 1.30882447817669 4.51530983705265 8.69506773644972

N 1.54982285250272 2.62657819668292 6.32513125730824

N 4.33169885574950 5.49350607531111 5.54138983893071

N 4.95179561616321 5.49627815857702 4.37454812303411

N 4.20196554429639 3.45803317915470 4.78206429749390

N 5.22141905366962 2.45518321471431 2.28152332515163

H 4.72606513283605 1.76330779016607 2.81618995750578

**[Cu(phen)_2_(HL)_2_]; multiplicity 2**

C 6.72999169271549 15.60775875604913 5.72565929200848

H 7.57340040288622 15.51495970697174 5.05454434979175

C 6.88076531154576 8.91478472106501 5.18586973211435

H 7.32472291444224 9.01323289110644 6.16772450108968

C 9.43622346582487 13.47600202063399 2.89800370892203

C 7.97596833222827 11.07816783450239 8.94545708248550

C 6.39889623976263 16.84431638328289 6.29620506777922

H 6.99749080458715 17.71458200972367 6.07184915412326

C 6.85483913319109 7.67567248297360 4.53183916156713

H 7.28983759610946 6.80966090879117 5.00802809228248

C 9.98349067971190 14.39556990577298 1.91345025535514

C 7.98513603922292 10.20796478595065 10.11049130792755

C 5.31204957242457 16.91837903384747 7.12559753102767

H 5.01819487281343 17.85429786036547 7.58184053251727

C 6.26836295570634 7.59396187180972 3.29716924487161

H 6.22193209025533 6.65582696753393 2.76043169503743

C 11.17151838918493 14.36857524978348 1.24017204452378

H 11.93513303936416 13.61672446786087 1.33973159128800

C 8.79885062649877 10.20922840207326 11.20740024886313

H 9.60941912568123 10.89342511617706 11.38951644322435

C 4.56271366162594 15.75793831521331 7.38713945605697

C 5.71593723422221 8.74919166048432 2.71685459965967

C 11.18924325766835 15.52357894290167 0.39061215644706

C 8.35974179497501 9.13312282815627 12.04693092179005

C 3.40638573207421 15.76194657766529 8.23729393332685

H 3.10774339573139 16.69611029489698 8.69312495367928

C 5.07883174365984 8.73828574821683 1.43072936772316

H 5.02754752207716 7.80226373456907 0.89145153664599

C 12.11062718623879 16.04610213952805 -0.52698788007160

H 13.05014219865552 15.54068266619252 -0.70811755043024

C 8.77917902458302 8.62771325052875 13.28490704602212

H 9.61221783739185 9.07960633039658 13.80716140870999

C 2.70925822385066 14.62991868428992 8.45546937555619

H 1.83411382634323 14.62736584677169 9.09072354726848

C 4.55535750558160 9.86664645218002 0.91313728926114

H 4.07059083550405 9.86446520410453 -0.05350156453955

C 11.79989976825099 17.21255155798190 -1.19319971929799

H 12.50148962711103 17.62753415466771 -1.90417474972028

C 8.11318145673962 7.54787241309147 13.82459758700549

H 8.42571204711293 7.14729887421704 14.77945951249930

C 3.11164167174028 13.39115586802210 7.85152982587017

C 4.62902693063493 11.10800826352544 1.63072669068992

C 10.58074335775743 17.87752948626394 -0.96527801722192

H 10.36726508440072 18.79073937845999 -1.50428444312208

C 7.02839184588981 6.95334789848484 13.15356967332004

H 6.52681501966472 6.10754532845931 13.60417730130404

C 2.40942548362170 12.19253476614779 8.06127269335618

H 1.53244297858005 12.19507960395999 8.69453931447736

C 4.09692908905540 12.30300139545102 1.11821326410845

H 3.60967883311816 12.29591246813103 0.15245088778944

C 9.65425293768534 17.38781043546341 -0.06874701496342

H 8.71632367626575 17.89611281862894 0.10923496158210

C 6.59294099698298 7.42814522569851 11.93413136767027

H 5.75957140530504 6.97351222388181 11.41589379747228

C 2.84561359393934 11.04166248678853 7.46152484840636

H 2.33245012687852 10.10180794831427 7.59741524062331

C 4.20369065012766 13.45640003628201 1.84815950998567

H 3.80680197841876 14.39364590359736 1.48870555268598

C 9.97345441658509 16.20721529104339 0.60287705991937

C 7.27054012252857 8.52067109770663 11.39195058568023

C 3.98984537182318 11.09270420863968 6.66070403914958

H 4.37005990738457 10.20066250750164 6.18508603852093

C 4.85167961063537 13.41162989479622 3.08565994274619

H 4.96691690387427 14.30589866438815 3.68033512882707

C 4.24005584926539 13.35190447067288 7.02478305695080

C 5.25077974934539 11.15371066274324 2.88344073248710

C 4.97959608263761 14.56945878665940 6.77700125898520

C 5.79994112035591 9.94049261494721 3.44614320073322

N 6.04532892448086 14.50702691465769 5.95419180943826

N 6.36951142465244 10.01004203049756 4.66543485675503

N 8.13669714237005 12.60446674992049 4.27959668253208

N 7.45592660292298 11.91104978737432 7.10351903884406

N 4.66492042900880 12.20472650143231 6.44922493341629

N 5.35875326062531 12.30323149721067 3.58691825889643

N 9.20036717439804 11.84974969322057 4.22432069504667

N 8.44717385861326 12.61612653585952 7.57739579659087

N 10.04913285175456 12.37688511900047 3.35363147149905

N 8.80306957615434 12.11229837639036 8.74997677673770

N 8.24171290570948 13.64120043156267 3.45582733698780

N 7.12386572635451 10.93072084863655 7.93664035901441

N 9.25743923887054 15.49998322997507 1.53390558052424

H 8.34964043357448 15.73777638581899 1.89384943084860

N 7.06274129319841 9.19349495393612 10.21552037773284

H 6.35656903630735 8.99036468212912 9.52989203944587

Cu 6.44748916294783 12.25611447538869 5.36728379326347

**[Cu(phen)_2_(L)]^+●^; multiplicity 3**

Cu 2.76425079155380 3.77508429819707 7.42670961122893

C 5.25716777499586 4.94437428963410 8.62616154784222

H 5.75189393762877 4.46714939563539 7.79286623668060

C 3.69473159950196 1.46922454460524 9.48005649019142

H 4.36979718676853 2.20947138840228 9.88886887815344

C 4.81074337613955 4.04722925645690 3.93774477987528

C 5.98359158792348 5.65154430933510 9.58957419187896

H 7.05661938237919 5.72015696028443 9.49721356975926

C 3.62157030445028 0.18588493661781 10.03747037738975

H 4.24664598494671 -0.06399843964029 10.88142067343522

C 5.36042376588447 3.53230499493063 2.68918173934993

C 5.31553948147560 6.24424984545195 10.62946510004085

H 5.84921458614665 6.79909358260396 11.38923114218140

C 2.75644022359753 -0.72691769270831 9.49402371103020

H 2.67600852562372 -1.72743177758027 9.89715074395495

C 6.10263864178548 4.34439641207021 1.74220762781903

H 6.33298829104849 5.39165188559622 1.84465656950850

C 3.91679573678996 6.12831166657524 10.71121479573328

C 1.96123321219517 -0.35639773211420 8.39493033328741

C 6.43741433268480 3.47110062248557 0.69640029289021

C 3.13297904536359 6.71072721829117 11.76453424107686

H 3.64627621856574 7.27066342237105 12.53396421575990

C 1.03131355828390 -1.24785965396352 7.75988099989083

H 0.93910287221670 -2.25307361945467 8.14765461224037

C 7.13703946406490 3.61796557577096 -0.50746210805865

H 7.55519141970731 4.57363745464903 -0.79278501403696

C 1.79239462499954 6.56170360931348 11.79361776672914

H 1.20282809917588 6.99948693433231 12.58722037240405

C 0.29526268863930 -0.84321374246603 6.70479982626671

H -0.40223456543045 -1.51437326300694 6.22305552628163

C 7.27751605532310 2.50514841753137 -1.32164172513555

H 7.81194378199834 2.58600209055860 -2.25756032963778

C 1.11111877547985 5.81730425397891 10.77156197568415

C 0.41734398771148 0.49230701778763 6.19078569865372

C 6.73433955599331 1.27526687037926 -0.94614579326458

H 6.85885531110734 0.42359400429860 -1.60095661046479

C -0.28177621043611 5.62497252518724 10.74891440130049

H -0.89239143428445 6.05236080809489 11.53273942847566

C -0.32361624321153 0.96429538955773 5.09389680906273

H -1.02439331118807 0.30381026919471 4.60146450061305

C 6.03201528439063 1.11263552358419 0.25471281728267

H 5.61647373252764 0.15388003940776 0.53212641268313

C -0.84403233724287 4.89740066758507 9.73306700266243

H -1.90908418321306 4.72917559808210 9.68352896253138

C -0.14726834321688 2.25288352007086 4.66038529493599

H -0.69676689073472 2.64605597542871 3.81888672271061

C 5.89062026926280 2.21262729479736 1.06469161054249

C -0.01748056912616 4.36169982571682 8.73776054712204

H -0.43735419824243 3.78356896600165 7.92666311069791

C 0.76589021386539 3.07325951915680 5.32765217186789

H 0.93203942985419 4.09494330634346 5.01554554794914

C 1.84994532577157 5.24502672552523 9.73645001033550

C 1.30970540385026 1.37961549448325 6.79492730365633

C 3.27614811294934 5.40336379398429 9.70737099443135

C 2.10371749977276 0.94646522458268 7.91448670133110

N 3.94496574046540 4.82497633866859 8.68436763192424

N 2.96264936213000 1.83907719737517 8.45042538544565

N 3.84519678014330 4.21001330704527 5.77598880679989

N 1.28913673177200 4.53031494777584 8.73645444448962

N 1.46444979144226 2.65143067295400 6.36421168732518

N 4.30321199818391 5.40316911969342 5.47648941992838

N 4.92094435936490 5.32996859239211 4.31435793524256

N 4.14226147906413 3.32797519921909 4.83365019474949

N 5.22875258936578 2.29032482088125 2.30979207728263

**[Cu(phen)_2_(HL)(L)]^●^ ; multiplicity 3**

C 6.77398287109059 15.59483204026601 5.83091352644029

H 7.62199587444493 15.51152101204065 5.16418900032242

C 6.88675589577607 8.92552293470201 5.11156676054928

H 7.31098459789800 8.99702705647874 6.10426191282132

C 9.45133430472252 13.50148312327472 2.93281123408155

C 7.83341971600796 10.94831043921513 8.95579096403314

C 6.45860728292512 16.81652803646759 6.44106360444320

H 7.07286226635333 17.68403551361861 6.25100632123520

C 6.87601442156901 7.70472917973072 4.42351468491402

H 7.30370432310181 6.82685243310697 4.88437246014565

C 9.98110157888102 14.39816646624929 1.91792915609463

C 7.80270045332475 10.06281134375555 10.11330940023456

C 5.36709481866552 16.87987206366405 7.26536277220784

H 5.08547442921238 17.80431755941467 7.75173718727647

C 6.31307431203224 7.65565309656057 3.17628850383276

H 6.27840796903443 6.73229705784809 2.61366343370547

C 11.18723708159891 14.39819916811855 1.27698788495859

H 11.98583365853816 13.69471275600351 1.43705549191293

C 8.70309936775765 10.18075376802922 11.24685203506016

H 9.48342116222904 10.91347464589475 11.36750609672609

C 4.59725879685058 15.72360046868809 7.48216606383257

C 5.76920294182830 8.82545010055294 2.61743854891634

C 11.16812513701403 15.50424825181516 0.36454093544148

C 8.32820808249557 9.14872885214879 12.12033630167329

C 3.43601412238233 15.71601214381771 8.32563377569547

H 3.14917231377820 16.63819992135138 8.81237499312117

C 5.15495562323733 8.84842256021342 1.32039994959847

H 5.11550577188295 7.92721998575220 0.75520857214721

C 12.08504146100368 16.02327671040881 -0.55952110927339

H 13.05449254950275 15.56020448947391 -0.68858275929330

C 8.78563143524415 8.71804874195850 13.37162829950284

H 9.60650546815975 9.22029987029410 13.86503770251000

C 2.72053796921110 14.58788722548115 8.50086623068688

H 1.84220038964889 14.57689649053390 9.13161295495391

C 4.63734461870173 9.98923771857913 0.82465143262952

H 4.16919467202004 10.01200296517236 -0.14984523720406

C 11.73083407222076 17.13083508655482 -1.30035303129564

H 12.42824742208109 17.54187238051047 -2.01770486812614

C 8.15711085225791 7.63162496642640 13.95923575129772

H 8.48707860482976 7.27548048184458 14.92485456077290

C 3.10686085479829 13.36466617236733 7.85596094338292

C 4.69529283347326 11.21080676194340 1.57689007498839

C 10.47236373245357 17.74031940384330 -1.14110046101732

H 10.22511452250547 18.60777798260922 -1.73811560649858

C 7.09800332007117 6.98844877013198 13.31603997444414

H 6.62567569119649 6.14303899895860 13.79760829546185

C 2.38612930377049 12.16987648172098 8.02032026632216

H 1.50648475266436 12.16305418734461 8.64984378763812

C 4.16787078031744 12.41807102586318 1.08897492692410

H 3.69648936496475 12.43629609244642 0.11550995586410

C 9.54955217026464 17.25321383524763 -0.23937546953085

H 8.58128940993854 17.71859957173916 -0.11432747594854

C 6.62932074485030 7.40709219277477 12.06464996953778

H 5.80767170933420 6.89947354440127 11.57871275535572

C 2.80787499726237 11.03442880768939 7.38200224274610

H 2.28031926923941 10.09817293994726 7.48292442477646

C 4.25842119364771 13.55117047708111 1.85233557514661

H 3.86385721283374 14.49664969881363 1.51242972489892

C 9.91282332155318 16.13220322416291 0.50779590581872

C 7.24930326530298 8.48348785492945 11.47842554035946

C 3.95698749430349 11.09608687885835 6.58888003730972

H 4.32724210021824 10.21574613495859 6.08426678008198

C 4.88657325774327 13.47420722470100 3.09837967371377

H 4.98973317185788 14.35195744230054 3.71950942508445

C 4.23889101479283 13.33706912792658 7.03410486878535

C 5.29602346951393 11.22370590385966 2.84056359379223

C 4.99897525209596 14.55045913676961 6.83365614668190

C 5.83756454809351 9.99659387907454 3.38004031616755

N 6.06952388670707 14.49872301938974 6.01647941989248

N 6.38345283679830 10.03388996959310 4.61151277135094

N 8.15745702789208 12.63858045581752 4.32531175304372

N 7.40982667766136 11.84614902432688 7.12323543306338

N 4.64950179467397 12.20458653179101 6.42060171674880

N 5.38947483105387 12.35383225515462 3.57643029984340

N 9.24388314158398 11.91470336342618 4.31082060686038

N 8.39426701658380 12.52216655100088 7.66279457453201

N 10.08962014413348 12.43723603475369 3.43462699858690

N 8.68644497961646 11.97444154699724 8.82890433743894

N 8.24468970343795 13.65001163014360 3.46820739324685

N 7.02546409485442 10.84951255393032 7.90471686170823

N 9.20975520550332 15.44029228547287 1.45987017404369

H 8.28007426041800 15.64644540569048 1.78128707246441

N 6.95202084880707 9.07986952791753 10.23399297565573

Cu 6.44884610169843 12.26272698611182 5.37116992064960

**[Cu(phen)_2_(L)]^2+^; multiplicity 3**

Cu 2.82873229778688 3.59551256003434 7.45821261272536

C 5.31374224475312 4.67039617092431 8.73371731850977

H 5.81862326428462 4.10910385326254 7.96036184732880

C 3.61484736020891 1.42878143109021 9.65635625936485

H 4.28787428309655 2.17818822920507 10.05009321676250

C 4.85070236336085 4.11704271268416 3.94929009049351

C 6.03465065480007 5.38173617134709 9.69717033456683

H 7.11333064609545 5.36924564796921 9.66649525566121

C 3.49168693332362 0.18200951133276 10.28283941940401

H 4.07691480183167 -0.02868419733728 11.16511686911016

C 5.36932581876303 3.68850387216378 2.68636286677592

C 5.35343850597522 6.08006509531785 10.66038023445585

H 5.88230946329102 6.63977851108077 11.41993772196763

C 2.62961588349841 -0.74507697348329 9.75893155226926

H 2.51149759570403 -1.71759815839209 10.21733139098687

C 6.09075485866688 4.47868415673429 1.73369438554800

H 6.33524167854147 5.51875228468055 1.86314967954626

C 3.94775566554300 6.06850191613489 10.66278960270153

C 1.88725625558234 -0.42538502029560 8.60803577576048

C 6.38428524435220 3.64250137780627 0.66581757878843

C 3.15278129735880 6.76742886886289 11.63365121619690

H 3.66349480409909 7.33415449761579 12.39979557266986

C 0.96077722671644 -1.33151028147510 7.98877779115807

H 0.82925639568553 -2.30997761539022 8.42982854233306

C 7.06543046677789 3.84054119991000 -0.55841822623000

H 7.47774202428732 4.80938531867002 -0.80075016651886

C 1.80541869546004 6.71543866375031 11.59137096636390

H 1.20699826187469 7.24001711945158 12.32330644150701

C 0.27366116568996 -0.97197969872061 6.88515151249705

H -0.42300650531341 -1.65384346053459 6.41750566915525

C 7.18145772381560 2.77316777655362 -1.41459486867954

H 7.69483256914626 2.88809187280098 -2.35769691685126

C 1.12832020307079 5.96130774256941 10.57364047543390

C 0.44781678496990 0.32817042591683 6.30015453341008

C 6.63761167096165 1.52135278843949 -1.08083226864331

H 6.74696816146972 0.70221516325759 -1.77764276050081

C -0.27184034810504 5.86586851115248 10.48210102296204

H -0.89010281905048 6.38160917511779 11.20434190562275

C -0.24228447327178 0.75735302420411 5.15341749777832

H -0.94425521650212 0.08919796452109 4.67318243493271

C 5.95906032321968 1.29224218956714 0.11951356337601

H 5.54745989546548 0.32308206539264 0.35928500481113

C -0.83110298397414 5.11799284922176 9.47948923904498

H -1.90147952509345 5.02032896585305 9.37998459149750

C -0.01680379858013 2.01568336057200 4.65787826178640

H -0.52874000973437 2.37639782508160 3.77900229778335

C 5.84710923457072 2.36065474101454 0.96862832331467

C 0.00637520946310 4.46718895242272 8.56488872745327

H -0.41062054224595 3.86963980820088 7.76616974133964

C 0.89705429308425 2.84860100832447 5.30881238680564

H 1.09952851254343 3.84700343059737 4.94728176675023

C 1.87739349200594 5.27731069326174 9.61628645580355

C 1.34165616657548 1.22568293694036 6.88491528814660

C 3.31162278733618 5.33148139295063 9.66423473058044

C 2.07826695659958 0.84251186289629 8.05806228579496

N 3.99471797191604 4.64533076373047 8.72001526143208

N 2.93291997996783 1.75044860068431 8.57673655914028

N 3.90084113153761 4.13023863002977 5.79472985061947

N 1.31979939626010 4.54553929669724 8.62699999507793

N 1.55093787956178 2.46615797641695 6.38920094582763

N 4.37534683179197 5.35031357502802 5.58301754549827

N 4.97944153736089 5.36749413355876 4.42356118306887

N 4.17991849597450 3.33120468259893 4.79103072015862

N 5.23666442333027 2.45006197091768 2.22043749761582

H 4.76595743246167 1.69771907910600 2.70175638994568

**[Cu(phen)_2_(HL)_2_]^+^ ; multiplicity 2**

C 7.00338687944324 15.24802594289428 6.04709287755295

H 7.90047589030342 15.14051023333940 5.45787986693276

C 7.28800266387114 9.38065750636073 5.12767977657007

H 7.78954184052417 9.49671989004894 6.07543125043885

C 9.42792767727114 13.58470428483895 2.93449672419676

C 7.92730839258694 11.03113951025290 8.99841297736044

C 6.67191012965436 16.46639895316252 6.64900816259192

H 7.33280833938583 17.30978245081496 6.52145790775737

C 7.30865727302932 8.16288232688615 4.44011433350121

H 7.84203085309114 7.32872980435270 4.86911360949545

C 9.91082160232737 14.40886329054406 1.83983908965548

C 7.86170444739708 10.18322525602014 10.17642818298175

C 5.51893419298056 16.56437407449354 7.38301047311217

H 5.23807195454697 17.49590623641257 7.85519124820862

C 6.65255905067973 8.05361874137803 3.24200264678756

H 6.65124050397675 7.12231925569754 2.69231623676735

C 11.12018449192946 14.40783676880207 1.20590398639291

H 11.95276095725640 13.76630385491701 1.43720009659831

C 8.64832140835428 10.16643461937016 11.29233478685967

H 9.48896604554424 10.81144577762678 11.48096795152145

C 4.69020567986526 15.43751381905561 7.52852845794743

C 5.97570159245872 9.16864884706323 2.71637993714674

C 11.04984082221080 15.41523420734140 0.18862211423653

C 8.13281218955209 9.13284963847879 12.14118775071518

C 3.46901103412283 15.43745194465171 8.28290574569351

H 3.16397267646330 16.35753866901269 8.76125152693655

C 5.26916395664408 9.15725391676414 1.46668181312970

H 5.24829659116918 8.23662236685404 0.90057169083475

C 11.94653744932991 15.88638584626704 -0.78035833068079

H 12.93913817422865 15.46310758730714 -0.86001048744595

C 8.49606451407634 8.63302609537023 13.39951922537134

H 9.33905378454851 9.05213380292661 13.93286469370171

C 2.72253230972162 14.31857201149886 8.39334994821051

H 1.80328542829654 14.31843644647457 8.96209747218129

C 4.65031117828957 10.26627800595384 1.01000935007831

H 4.12015592210355 10.25825234183770 0.06805928111678

C 11.54163934836458 16.89504355862856 -1.62759506618113

H 12.22204679639172 17.26892143032137 -2.38060760113561

C 7.76186544166784 7.60125987765865 13.94318885903344

H 8.02967581419443 7.20496538214631 14.91323379785904

C 3.13020822247363 13.09555483465859 7.76218153280079

C 4.68340169450452 11.48994150529544 1.75968558714057

C 10.25209415260234 17.45194369635422 -1.53161332546067

H 9.96566598343949 18.24245544005836 -2.21205922146039

C 6.66290067123806 7.05012116663783 13.25703424408085

H 6.10703720280883 6.24160166912490 13.71232013093942

C 2.40620691102489 11.89309223213087 7.84660543094262

H 1.48370936255263 11.86510318883216 8.41014319733681

C 4.07377786048711 12.68390613481644 1.33529788555215

H 3.53874323841752 12.70425814473575 0.39575950580185

C 9.34854438151662 17.01006279341708 -0.58892451199516

H 8.35704445032897 17.43522717919911 -0.51288539681368

C 6.28174780451494 7.52016209903147 12.01840651605190

H 5.43797378957517 7.09899569227631 11.48897688352823

C 2.88016825191987 10.77296183466389 7.21528697582796

H 2.35123019180432 9.83350575684996 7.26025113432099

C 4.16889149057854 13.80622871485618 2.11591840315727

H 3.71607762273260 14.73975884570101 1.81937290831445

C 9.76310250061323 15.98788972615548 0.26642667186346

C 7.02913973063374 8.56440903011307 11.47147781736098

C 4.07850457611062 10.84445501955644 6.49737704687330

H 4.47641314262443 9.97334882435436 5.99919901871857

C 4.87221273435679 13.74531735012848 3.32342202924231

H 4.97049673630872 14.61867850922239 3.95039366732151

C 4.31075347935727 13.07680587293555 7.02239832907015

C 5.35873324896489 11.51884224439060 2.97803371325385

C 5.09726077740599 14.26290597921902 6.90008603673586

C 6.00868005239463 10.34338163948454 3.46404936284097

N 6.22991371538669 14.18723666921766 6.16435840160162

N 6.64615306465820 10.43010636086866 4.65394043596250

N 8.20048988490135 12.78018209124559 4.40238816454364

N 7.51238670039994 11.85626621805165 7.13947158529382

N 4.76496227271278 11.96404116899559 6.40641372192682

N 5.44281802696176 12.63317023426430 3.73606976706550

N 9.31972066152537 12.10983994210393 4.44754444661984

N 8.51148704244832 12.53686969119431 7.63156401453115

N 10.12527258522249 12.60142121380165 3.52704437987203

N 8.80300788502187 12.03284221900683 8.81457208585587

N 8.21701633561732 13.71392755821046 3.46093410733758

N 7.10394637095725 10.90324674964483 7.96593580352166

N 9.08849351082079 15.35791920941712 1.27989686853233

H 8.14735792214183 15.54890016857258 1.57639569616674

N 6.88482486928664 9.22073640619804 10.27675726775952

H 6.18295153449516 9.04073233149987 9.58031690629785

Cu 6.54490305829742 12.30911706967911 5.43895040973111

**[Cu(phen)_2_(L)] ; multiplicity 1**

Cu 2.88840401398186 3.53892366407357 7.49653152138437

C 5.48966408355208 4.61223319492358 9.39409910495898

H 6.07837075489471 3.95837906294775 8.76184034411233

C 3.75260087187966 1.23475727874767 9.32653193367716

H 4.42160111896138 2.00714576273739 9.67960312180666

C 4.88448864765088 4.24514406542752 3.97115177454369

C 6.10948184250622 5.36443279915060 10.40073186770508

H 7.17742335006033 5.29377826336577 10.54510330329470

C 3.77006436210648 -0.03533395989336 9.91322522435619

H 4.45739352733908 -0.24225390406729 10.71993339714396

C 5.40003988525891 3.84393078322376 2.67114722315592

C 5.33216177439696 6.17341363955461 11.18625899044584

H 5.76506555125741 6.76766639324338 11.98004794334855

C 2.90891498630149 -0.98928821739536 9.44113474253060

H 2.88974384443382 -1.98560667278438 9.86223371509278

C 6.09451839403286 4.55927487708347 1.73676337530003

H 6.38529930887098 5.59191363905047 1.82288860873225

C 3.94746850036309 6.23382586718094 10.95545538034293

C 2.03521756104745 -0.66628039693258 8.39000639448619

C 6.35144684086418 3.66919519497733 0.64256979217503

C 3.07516748750199 7.06058553527071 11.74098486356475

H 3.50752622759913 7.65056827549828 12.53796280264041

C 1.11292984066899 -1.62382341684696 7.84619803794643

H 1.09998137583166 -2.61843329512294 8.27119441282556

C 7.00670830477701 3.79971009069992 -0.58929338784071

H 7.45276909685471 4.74331777377780 -0.87518501286256

C 1.75227727056836 7.09590936516718 11.49058167282253

H 1.09002457155454 7.71474224003751 12.08075601561764

C 0.29178277163570 -1.29198719485994 6.83157458701594

H -0.40020276951468 -2.01235019626869 6.41650854432566

C 7.07398929847193 2.70756223135572 -1.42852505152812

H 7.57690755533410 2.79409797532192 -2.38225245451047

C 1.18355961438165 6.31607004113935 10.42687654971468

C 0.31315554305389 0.03140952699405 6.27517809083886

C 6.49776984871399 1.47603154412433 -1.06645523024415

H 6.56747973743849 0.63841770252885 -1.74731898063848

C -0.19071815511666 6.34163620785361 10.13787110023652

H -0.84821681756531 6.96091403375672 10.73327803720836

C -0.52758596676855 0.41293788753867 5.21601210356634

H -1.21695409995723 -0.30880767174727 4.79817461370429

C 5.84574215533790 1.31721375488859 0.13881113733620

H 5.40111857627257 0.37237044976952 0.42056812338546

C -0.67214351261623 5.58112807086616 9.10617140643043

H -1.72149866018884 5.57124609766397 8.85151197772737

C -0.45831354010343 1.69172073183629 4.73041784577160

H -1.08599656635096 2.02147493071893 3.91576471791017

C 5.78074353032953 2.42478282226769 0.98443419176580

C 0.22715285178771 4.80454523806528 8.36721846289624

H -0.12492759866948 4.19661612492933 7.54547618623683

C 0.44903954678789 2.58306665378605 5.31837103247339

H 0.52102003512666 3.60295880012030 4.95957692471712

C 2.00505378656865 5.50092346368398 9.63668467557606

C 1.19186815000541 0.99561646543213 6.78807934436217

C 3.42623186445447 5.44955944814083 9.91734551051446

C 2.08667307389352 0.63398805268590 7.86985251263668

N 4.19495789570714 4.64387635277275 9.15926515787463

N 2.94564784302896 1.56778794456807 8.33723069638071

N 3.93708702470729 4.17502859751724 5.84317811890271

N 1.52251004860827 4.76084924708594 8.61333669018922

N 1.24390847475420 2.25555982397338 6.31498063038610

N 4.39926977893514 5.38828580373166 5.67673530438953

N 5.00737880984316 5.46643176884285 4.49701290029671

N 4.22575706499734 3.42054174647816 4.78191699663378

N 5.20765162437056 2.55837086191068 2.22298988493553

H 4.72333678718770 1.84265575742920 2.73598349727165

**[Cu(phen)_2_(HL)_2_]^-^ ; multiplicity 1**

C 6.73139904953928 15.69119230142544 5.73209348750009

H 7.58073597738980 15.62798477302006 5.06566158889480

C 6.90634040479558 8.90667764861189 5.24409902215707

H 7.34603318628955 8.98103786453613 6.22926125672231

C 9.28347396651984 13.39413552063412 3.07513699536947

C 7.86125920830978 11.20629865635676 8.78590231822126

C 6.37984276969670 16.90727550110740 6.33500317233852

H 6.97146283734002 17.78915829498229 6.14053964321284

C 6.89054312290778 7.68608562919324 4.55442750155614

H 7.33304638982282 6.81187498626443 5.00770671633588

C 9.87770188237221 14.28216623074408 2.09229604639833

C 7.93962358026208 10.31619188112429 9.93017110632121

C 5.28264268334986 16.94988115971264 7.15207094827924

H 4.97305105003514 17.87094226484792 7.62708402785186

C 6.30181264488003 7.62928348363541 3.32012250519290

H 6.26026074067697 6.70417262322356 2.76136515005706

C 11.08857373645049 14.21406369514643 1.46565768736274

H 11.83490259791794 13.45337963467512 1.61611888907946

C 8.74011859037039 10.37770721063389 11.03422258378147

H 9.47995609988375 11.13294902018202 11.23548797456981

C 4.54083744696353 15.77542296544749 7.37469707864169

C 5.73864231934484 8.79457636009442 2.76876011182160

C 11.15381378479490 15.33950070023519 0.58048057574582

C 8.39448913751547 9.25296901462289 11.85238096398073

C 3.37035793006572 15.74117060528897 8.20144662435859

H 3.04929064668098 16.65769988106909 8.67658554880425

C 5.09398563698666 8.81529031433756 1.48855571101320

H 5.04205654998490 7.89458878373456 0.92416751115026

C 12.11557394288032 15.81520965688408 -0.32190738023235

H 13.05218690656272 15.28942589870762 -0.45203626909968

C 8.84767364173268 8.77133744517067 13.08854533012933

H 9.62977848840372 9.29136751654146 13.62563926992484

C 2.68463105060077 14.59420948329589 8.37365589025909

H 1.79683475341120 14.55963673417765 8.98931892274123

C 4.56262003606929 9.95548592578266 1.00593130834968

H 4.07060066331491 9.98074471213658 0.04385704671783

C 11.84598091161067 16.96083623196630 -1.03828327617175

H 12.57738486088295 17.34048637934720 -1.73870782126378

C 8.27977244196245 7.62735035127179 13.60518145586398

H 8.61790658504762 7.24321273585041 14.55794466326362

C 3.11684216826255 13.37775319956671 7.74794825846689

C 4.63974457900525 11.17741238108480 1.75360724971590

C 10.62935836287467 17.65121315622327 -0.87621490828299

H 10.44929543300311 18.54741578221503 -1.45444711005320

C 7.26085759417690 6.94454428924314 12.91347071288470

H 6.83573745244219 6.04956644281923 13.34730622843076

C 2.43058055941908 12.16459006990876 7.91876259651095

H 1.54026403910978 12.14352068692901 8.53222490582583

C 4.11005588373488 12.38451564874464 1.26978516140744

H 3.61688485802319 12.39690717319607 0.30744265400164

C 9.66494022867135 17.20846255791935 0.00306712848052

H 8.72955120220682 17.73620131555075 0.12978382638117

C 6.79492289778203 7.39331143215784 11.69661201456100

H 6.01243030642292 6.87125444280114 11.16304935009186

C 2.89503604226402 11.02749681571029 7.31377051135780

H 2.39608846938712 10.07730764401676 7.42268886796893

C 4.22728411006331 13.52743261513948 2.01460051251264

H 3.83586580897022 14.47369368524208 1.67525181392997

C 9.94247838546593 16.04697888926512 0.72599120326475

C 7.37359634953726 8.55259359549089 11.17717994139846

C 4.05325003977228 11.09846922198327 6.54370777959342

H 4.46580327914589 10.22103984655456 6.07382305770568

C 4.88130512779603 13.46782767314634 3.24260661798616

H 5.01576424389838 14.34941490910862 3.84733368961187

C 4.26242063949859 13.38344980862578 6.94599761896458

C 5.26949233038684 11.18387751191453 3.00226688124725

C 4.98339639587278 14.61146815434234 6.74030872282007

C 5.81998532836085 9.96388791475284 3.52960122741060

N 6.05609168537686 14.57897301242636 5.92578353761657

N 6.38582857636072 10.00959626862118 4.75137339855756

N 7.94126285440086 12.55397422508056 4.39803102232164

N 7.28452225280451 12.04664631252893 6.99177898190841

N 4.70137181721632 12.23556348097219 6.36776115287444

N 5.37422040835190 12.33646064032700 3.71281652150915

N 8.99396068275394 11.78322343658369 4.42302400285626

N 8.22192732069282 12.82303302795441 7.46248132619811

N 9.87016581886778 12.29637423935780 3.58694084943490

N 8.61038275237643 12.31057117621341 8.60959802923692

N 8.06649797737606 13.57508231074235 3.56974137025476

N 7.01665822628954 11.02127360539235 7.78046120174297

N 9.18152160092518 15.38352982715854 1.65284129789642

H 8.26444986905957 15.64853330083090 1.96775709447947

N 7.11338073629306 9.21973721354682 10.00846400681842

H 6.43623895930815 8.95642866136113 9.31366144808593

Cu 6.34443909236461 12.29325732122801 5.37225435681179

**[Cu(phen)_2_(L)] ; multiplicity 2**

Cu 2.77625018354262 3.85397556728622 7.44188306308967

C 5.24408825739663 5.03813653583812 8.68291637709738

H 5.74990436098439 4.58841322302071 7.84121179103196

C 3.72022712575385 1.49278824095039 9.44430385874453

H 4.36612377305423 2.23637092758220 9.89276207577137

C 4.83780111757805 4.11588527900906 3.97421832295656

C 5.95293839675041 5.73944128031763 9.66390812908461

H 7.02490675393295 5.82994047169457 9.57805244428165

C 3.67971453475325 0.18598712945249 9.94813699424252

H 4.29975236834358 -0.07908607357845 10.79118889769816

C 5.36840864974950 3.58437839479526 2.71823313024857

C 5.26936582503630 6.29990318434320 10.71146682147847

H 5.78918993211862 6.85038175922035 11.48389913014599

C 2.85323860549598 -0.73034987524283 9.35295915092032

H 2.79940793953815 -1.74921384254638 9.71233409427594

C 6.11125767660469 4.30188973984257 1.78603832902121

H 6.40828620828386 5.33626344712410 1.85068757513855

C 3.87263232815456 6.15607619849656 10.78433057926246

C 2.06368689098584 -0.33976476558583 8.25685616878338

C 6.38481720597604 3.37667884226106 0.74609202006795

C 3.07247250105477 6.70393074225090 11.84386600571342

H 3.57102865140900 7.25981174582600 12.62586360326660

C 1.17584441143984 -1.23468802447803 7.56878696052324

H 1.11153856588102 -2.25772591851095 7.91321733857726

C 7.07378071981295 3.42904565827594 -0.47919793856011

H 7.54916156936525 4.34697387597652 -0.80558820975569

C 1.73484818419982 6.52862878825717 11.86324733168026

H 1.13305054911496 6.94082828250752 12.66136502907894

C 0.44569438261247 -0.81154243893699 6.51699893644278

H -0.21927973723038 -1.48509680589545 5.99429882755469

C 7.13901101578880 2.29775458999079 -1.26422571652213

H 7.66679547447557 2.32734257665336 -2.20912250229076

C 1.07307641590258 5.78921655239903 10.82478579708385

C 0.53116415437777 0.54750593669959 6.06041104866234

C 6.52518385938429 1.09369456113593 -0.85363128692865

H 6.59320638657216 0.22167776542771 -1.49196856062662

C -0.31547831176911 5.56944212708185 10.79149571615591

H -0.93845493274556 5.97078911910912 11.57932015363371

C -0.20350702728446 1.03742742026952 4.96728136318966

H -0.87024288926740 0.37343564733498 4.43402221194609

C 5.84365573002500 1.01477650590522 0.34190416720647

H 5.37355832742880 0.08896997691234 0.65281605449137

C -0.85817107089999 4.84834839186113 9.76047532945451

H -1.91939934098193 4.65987631075214 9.70276323016857

C -0.06285697140637 2.34735570631774 4.58838903148724

H -0.60759525333606 2.75470682538209 3.75052655943790

C 5.76442732261519 2.15615136261094 1.15906984923193

C -0.01646207105580 4.34668637372233 8.76018665890620

H -0.42032550831793 3.77399085364174 7.93716918540182

C 0.80814083773297 3.17029574493113 5.30694557649861

H 0.94771279588611 4.20776806095258 5.03680219474421

C 1.82796257561329 5.25050354744085 9.78332745719379

C 1.38043587516059 1.43948836156603 6.71788011863425

C 3.25085768279685 5.43804093881323 9.76396780989418

C 2.17030033561896 0.98638950156796 7.83337425741863

N 3.93436992035212 4.89353728885564 8.73269182039206

N 2.99212539031751 1.88209198189112 8.41928187881173

N 3.87775321649373 4.29070338927313 5.82793824729317

N 1.28628895435915 4.54226076828105 8.76826613194350

N 1.49909267349137 2.73158514522638 6.34113546036058

N 4.34830334182084 5.47383719684272 5.53383116940083

N 4.96248920859817 5.39746787979707 4.36527672094159

N 4.16205710215295 3.40605716494904 4.87508793006593

N 5.14532684840635 2.28962285685052 2.36626809845412

**[Cu(phen)_2_(HL)(L)]^-^ ; multiplicity 2**

C 6.70464199147441 15.58622454055636 5.68462147041877

H 7.52413439504765 15.49794115044593 4.98378521743399

C 6.82544476891886 8.88225503441341 5.16164678147086

H 7.26000825971547 8.98402582240896 6.14731852007229

C 9.43096154945858 13.45983357452351 2.87295159280680

C 7.88410923266974 11.03745311435024 8.89753547041554

C 6.41142350082429 16.81283485714534 6.29596346954666

H 7.01446998590191 17.68021675000219 6.07222053488608

C 6.79490404607072 7.64008094646117 4.51310727391504

H 7.22004191361931 6.77337329795000 4.99691772983032

C 9.99486401929932 14.39690473637427 1.91474575971111

C 7.88924720868733 10.16842887364991 10.07550154198278

C 5.35535629206338 16.88076704802083 7.16457836290224

H 5.09111213677808 17.80873813997949 7.65402754680193

C 6.21624742988058 7.55594501958448 3.27494947140724

H 6.16654942070150 6.61520731140684 2.74306447728032

C 11.18235724768041 14.36845668957499 1.24059221710230

H 11.93218278876753 13.60025397697432 1.31756382095696

C 8.77546445611470 10.25414036425083 11.14437389353637

H 9.58167555577078 10.96007382021245 11.26292552053828

C 4.59966281861831 15.72421740644698 7.42428733891030

C 5.67510496808073 8.71191216490188 2.68526503943664

C 11.22123786410370 15.54776651145834 0.42592040372047

C 8.38530942554382 9.21516919509546 12.02847453946011

C 3.47476088900634 15.72347533330534 8.31568756786942

H 3.20627342370974 16.65040702898527 8.80398407134526

C 5.04396568625323 8.69995275103889 1.39621363114074

H 4.98909943586842 7.76208377020206 0.86048124939251

C 12.15219365915567 16.08071573246976 -0.47573869953924

H 13.08235622582337 15.56384557432751 -0.67219809287695

C 8.83846091291104 8.74146820105462 13.27265279461344

H 9.68239578730669 9.20928571411765 13.76666369248314

C 2.77003410872446 14.59603351032505 8.53257673797530

H 1.91868927303251 14.58979397715485 9.19942850702076

C 4.52929336600170 9.82936022792887 0.87179697842328

H 4.04841552383980 9.82584558579147 -0.09677324217400

C 11.86316165845238 17.27232033818843 -1.10636448249667

H 12.57251696786011 17.69559078760377 -1.80463449336761

C 8.19649597707459 7.67264612715240 13.86178214941329

H 8.53756046227539 7.30078083550305 14.81976610494325

C 3.13347116706024 13.36670199518178 7.88629507791221

C 4.60670521937713 11.07324698686867 1.58475497140518

C 10.65662095291109 17.95255164473985 -0.85786209944354

H 10.46013870414409 18.88553373344227 -1.36871867807732

C 7.09535893594013 7.05166803014385 13.23242829282905

H 6.61093542840075 6.21468980603345 13.71962226673188

C 2.42339887852773 12.17265355597161 8.09562142294357

H 1.57084113973947 12.17193291173944 8.76144309199637

C 4.08219607071771 12.26937673171044 1.06717490380016

H 3.59808725146606 12.26197341952094 0.09983907430427

C 9.72104565195530 17.45297056632015 0.02373712262604

H 8.79269503712809 17.97292795435127 0.21760700273380

C 6.63086107242728 7.49578357375061 12.01257904258908

H 5.78532999452222 7.01640462482692 11.53282628927909

C 2.82182535154195 11.03030269779711 7.45507877370753

H 2.30206748065513 10.09390769483304 7.58989343984097

C 4.19144388872201 13.42429735585794 1.79449276120441

H 3.79951227621580 14.36211833870333 1.43114644415844

C 10.01810128875135 16.24690353676852 0.65907409813359

C 7.27069174490741 8.58333884312053 11.39315297002229

C 3.93842756117215 11.08496145182549 6.61611868327821

H 4.29144069148536 10.19793575658915 6.11093394832591

C 4.83483406262320 13.38038337961974 3.03449088004766

H 4.95152709370916 14.27479550367475 3.62877346562435

C 4.23109065440418 13.33147851035586 7.01869062923861

C 5.22410931414317 11.11986817933585 2.83936531317598

C 4.97800567820338 14.54524265349190 6.77142234760940

C 5.76332647230673 9.90587388929284 3.40912939672748

N 6.01305239467728 14.48918573016704 5.90994328204730

N 6.32579098435919 9.97803051971819 4.63130126814959

N 8.11353588944428 12.57267203407783 4.22819611958988

N 7.40267809079381 11.88950515995055 7.04443263551870

N 4.62075593252985 12.19220171003753 6.40517483354156

N 5.33532376756297 12.27098307499982 3.53998494890898

N 9.16932179279919 11.80797488381854 4.16175394870586

N 8.38509621267734 12.59109561912214 7.53747850948210

N 10.02924050704930 12.34460331465332 3.30709344915048

N 8.71582194870991 12.07710603570400 8.71405138495334

N 8.23483250034315 13.62571520986092 3.42759511959804

N 7.05318005341076 10.90380249843077 7.86596803265629

N 9.28898596805573 15.52496987419916 1.56834387592321

H 8.38629487357282 15.76879597996103 1.93706195153631

N 6.97280038569202 9.17080307956758 10.19937264785633

Cu 6.41379500207713 12.22339610852627 5.31850459094764
